# A zero density change phase change memory material: GeTe-O structural characteristics upon crystallisation

**DOI:** 10.1038/srep11150

**Published:** 2015-06-11

**Authors:** Xilin Zhou, Weiling Dong, Hao Zhang, Robert E. Simpson

**Affiliations:** 1 Singapore University of Technology and Design, 8 Somapah Road, Singapore, 487372

## Abstract

Oxygen-doped germanium telluride phase change materials are proposed for high temperature applications. Up to 8 at.% oxygen is readily incorporated into GeTe, causing an increased crystallisation temperature and activation energy. The rhombohedral structure of the GeTe crystal is preserved in the oxygen doped films. For higher oxygen concentrations the material is found to phase separate into GeO_2_ and TeO_2_, which inhibits the technologically useful abrupt change in properties. Increasing the oxygen content in GeTe-O reduces the difference in film thickness and mass density between the amorphous and crystalline states. For oxygen concentrations between 5 and 6 at.%, the amorphous material and the crystalline material have the same density. Above 6 at.% O doping, crystallisation exhibits an anomalous density change, where the volume of the crystalline state is larger than that of the amorphous. The high thermal stability and zero-density change characteristic of Oxygen-incorporated GeTe, is recommended for efficient and low stress phase change memory devices that may operate at elevated temperatures.

Phase change memory (PCM) has emerged as one of the most promising candidates for the next generation non-volatile memory applications. In particular, compositions based on GeTe alloys show high scalability to nanometric cell sizes, rapid switching speed, and good cyclability^1^,^2^. These materials are also being investigated for application in active photonic circuits^3^ and metamaterials^4^,^5^. PCMs, which are usually based on a chalcogenide alloy, manifest pronounced differences in both optical and electrical properties between the amorphous (disordered covalent bonding) and crystalline (ordered resonant bonding) structural states. Many of the prototypical phase change materials exist along the GeTe-Sb_2_Te_3_ pseudo-binary line. Considering in particular the reliability performance (retention/endurance) of PCM devices, two major issues have to be confronted for PCMs to be widely accepted as a universal non-volatile memory. Firstly, the glass transition, T_*g*_ (~150 °C), and crystallisation, T_*x*_ (~160 °C) temperatures as well as the crystallisation activation energy, E_*a*_ (~2.24 eV) of Ge_2_Sb_2_Te_5_ are relatively low^6^,^7^,^8^, which causes poor thermal stability in the amorphous state, and therefore limits its usefulness in automotive and aerospace applications. Secondly, as a general feature of phase change materials, there is a severe volume shrinkage (6.5%–9.6%^9^,^10^) and corresponding increase in mass density upon crystallisation, which applies considerable mechanical stresses in the PCM cells^11^, and consequently, causes resistance drift and void formation in the device that finally limits the cyclability of the memory cells. In order to address these issues, other elements including nitrogen^12^, oxygen^13^, silicon^14^, tin^15^, carbon^6^, and tungsten^16^ as well as oxides such as SiO_2_^17^and Ta_2_O_5_^18^have been incorporated into the Ge_2_Sb_2_Te_5_ material, leading to a higher crystallisation temperature with longer retention times and a reduced residual stress with smaller density change during the phase transition^10^,^18^.

Over the past decades, the stoichiometric composition of germanium telluride (GeTe) has received much attention as an alternative phase change material due to its simple composition and structure. GeTe is known as a growth dominated material that is capable of crystallising on a nanosecond time-scale^19^,^20^,^21^ and cyclability comparable to Ge_2_Sb_2_Te_5_^22^. At temperatures below 430 °C, GeTe shows a rhombohedral structure (space group R3m, No. 160) that can be visualised as a distorted rock-salt structure with Ge and Te atoms occupying the cation and anion lattice sites, respectively^23^. Moreover, the characteristics of the GeTe material have been tailored by element doping strategies, such as incorporating C^24^, N^25^, Bi^26^, and Cu^27^.

As a favoured doping element, it is surprising that there are no reports of deliberately doping oxygen into GeTe for data storage applications. Moreover, there are limited reports of environmental oxidation of GeTe films^28^,^29^. Therefore, the aims of the present work are: (1) to investigate the effect of oxidation on the fundamental crystallisation properties of GeTe and (2) to explore an hypothesised increase in the stability of GeTe-O materials, which could extend the use of PCMs to extreme environments. Hence, in this work the phase change behavior of GeTe-O films as a function of oxygen concentration is presented. This study has led to a GeTe-O composition with a unique set of phase change characteristics that includes a phase transition without volume change, which shows increased stability at high temperature. We believe that this composition is a promising candidate for PCM applications that require high thermal stability.

## Results

It is possible to increase the crystallisation temperature of GeTe by carbon doping^24^. However, carbon sputtering is an inefficient process due to its low sputtering yield. In contrast, sputtering GeTe in an Ar:O_2_ atmosphere is simple and a more efficient method to increase the crystallisation temperature. Furthermore, the reactive sputtering process is markedly dependent on the O_2_:Ar ratio; increasing it, increases the deposition rate, as shown in the supplementary materials.

**Figure 1:**
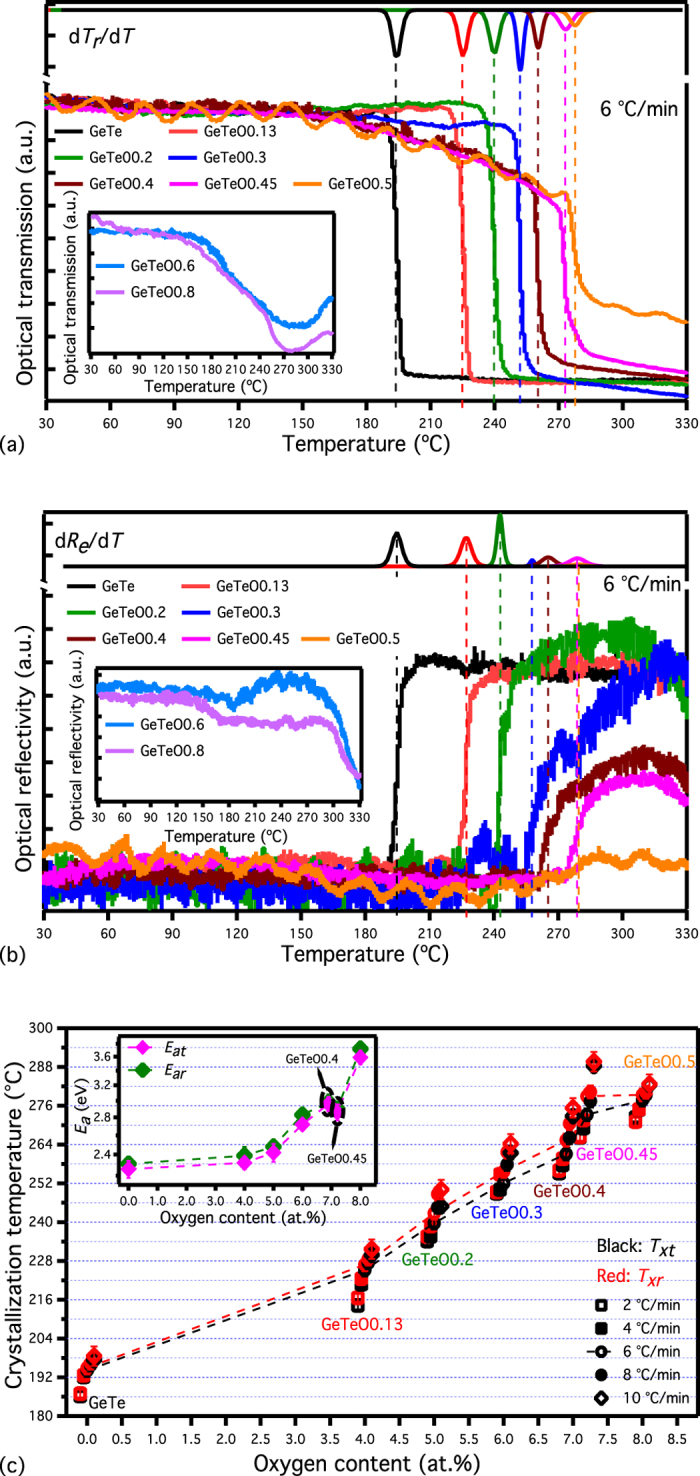
Temperature dependence of optical (**a**) transmission and (**b**) reflection curves of GeTe-O materials, measured at a heating of 6 °C/min. The transmission and reflection curves for higher oxygen concentrations are shown in the inset of (**a**) and (**b**), respectively. The upper graphics show the derivation of the transmission and reflection with respect to temperature (i.e. d*T*_*r*_/d*T* and d*R*_*e*_/d*T*) that determine the crystallisation temperatures; (**c**) Crystallisation temperature *T*_*xt*_ (black) and *T*_*xr*_ (red) of GeTe-O films as a function of oxygen content at different heating rates, and the compositional dependence of the crystallisation activation energy (inset). To aid comparison, a horizontal offset is added to each curve other than the 6 °C/min.

The crystallisation temperature of GeTe was found to increase substantially with increasing oxygen incorporation. The temperature dependent optical transmission and reflection curves of GeTe-O films are shown in Fig. 1(a,b). The measurements were made simultaneously at a heating rate of 6 °C/min. The amorphous GeTe-O films are characterised by a relatively high [low] transmissivity (*T*_*r*_) [reflectivity (*R*_*e*_)] at room temperature, which is stable with the increasing temperature until an abrupt drop [rise] in the measured optical response at the crystallisation temperature (*T*_*x*_). The samples with oxygen content up to 8 at.% [(GeTe)_92_O_8_] present a similar transmission/reflection transition profile, which is attributed to the amorphous-to-crystalline phase transformation. For the higher oxygen compositions, (GeTe)_91_O_9_ and (GeTe)_88_O_12_, the rapid transition is replaced by the transmissivity and reflectivity curves that are unstable with increasing temperature, as shown in the inset of Fig. 1(a,b). This is due to phase separation into GeO_2_ and TeO_2_ in the material. The crystallisation temperature *T*_*xt*_/*T*_*xt*_ in this work is defined as the point of minimum/maximum derivative of the recorded transmission/reflection curves with respect to temperature, as given in the upper panel of Fig. 1(a,b). The *T*_*xt*_ and *T*_*xt*_ of the pure GeTe film is 194 ± 1 °C and 195 ±1 °C, respectively, as shown by the dash lines in the figures, which agrees well with values reported elsewhere^25^,^28^. Oxygen doping substantially increases the *T*_*x*_ of GeTe. Doping GeTe with 4 at.% O increases the *T*_*x*_ by 30 °C. The abrupt transition from the amorphous to crystalline state for the pure GeTe material becomes smoother for the films with high oxygen content, which is clearer in the reflection transition profile, suggesting a slower crystallisation mechanism with additional oxygen atoms and therefore increased stability.

Doping oxygen into GeTe increases the resistance of the amorphous material to crystallisation. The crystallisation activation energy, *E*_*a*_, has been estimated by analysing the crystallisation temperature dependence on heating rate using the Kissinger approach: 
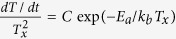
. Where *C* is a constant and *K*_*b*_ is Boltzmann’s constant. The Kissinger plots are determined from *T*_*xt*_ and *T*_*xr*_ under the heating rates of 2, 4, 6, 8, and 10 °C/min.

Note that the crystallisation temperature and activation energy measurements, which were established by analysing the film’s reflectivity, were consistently slightly higher than those established by measuring the transmissivity of the films, i.e. *T*_*xr*_ > *T*_*xt*_ and *E*_*ar*_ > *E*_*at*_, as shown in Fig. 1(c). Since the reflectivity is more sensitive to the film surface, and transmissivity is sensitive to the film thickness, we have attributed this difference to stress variation with film thickness. Indeed, stress is known to alter the crystallisation temperature of related materials^30^.

Generally, PCMs with a higher *T*_*x*_ and associated *E*_*a*_ show an amorphous phase with increased stability against crystallisation and thus an improved data retention capability is to be expected in high temperature PCM applications. The crystallisation activation energy of GeTe was found to be 2.26 eV inside the film and 2.31 eV at the film’s surface, which are in the range of values reported in literature (i.e. 2.0 to 2.72 eV)^24^,^25^,^28^. We suspect the reason for the higher activation energy is due to stress in the films. The activation energy of the GeTe-O films increases with oxygen doping, as plotted in the inset of Fig. 1(c). Hence, the data retention temperature (10-year lifetime) for GeTe-O materials is expected to be significantly higher than that of Ge_2_Sb_2_Te_5_ (~89 °C^10^) and pure GeTe (~97 °C^25^) materials. Incorporating oxygen atoms strengthens the amorphous GeTe-O structure by introducing stronger Ge-O/Te-O bonds. This accounts for the enhanced thermal stability of the amorphous GeTe-O materials. In fact, similar mechanisms have been discussed in oxygen doped Ge_2_Sb_2_Te_5_ materials, where the presence of nonstoichiometric Ge-O bonds is found to improve the structural stability of oxygen-incorporated Ge_2_Sb_2_Te_5_ films^13^.

**Figure 2:**
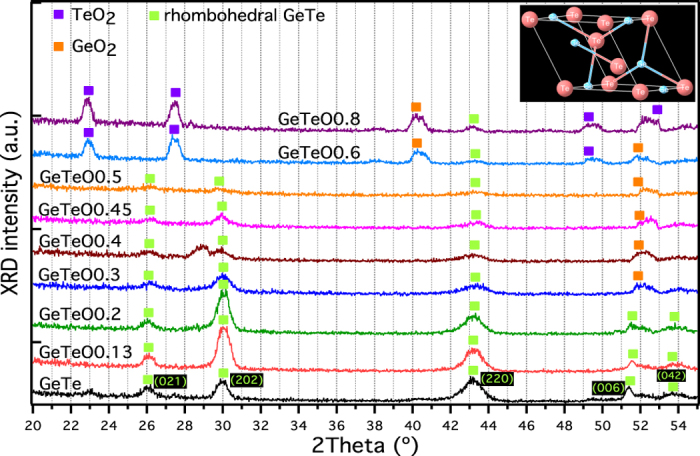
Typical XRD patterns of GeTe-O films after annealing at 300 °C for 20 min. A rhombohedral structure in the inset is identified for the crystalline GeTe-O films.

The XRD patterns for the undoped and oxygen-doped GeTe films annealed at 300 °C are shown in Fig. 2, and the as-deposited GeTe-O films are confirmed to be amorphous by XRD (not shown here). All the diffraction peaks in the 300 °C-annealed pure GeTe film are indexed well by the rhombohedral GeTe phase (JCPDS No. 47–1079), i.e. a distorted NaCl-type structure. For the XRD patterns of the oxygen-doped GeTe films, a similar rhombohedral GeTe structure can be identified to an oxygen concentration of 8 at.% O. However, at higher oxygen concentrations [(GeTe)_91_O_9_ and (GeTe)_88_O_12_], the rhombohedral structure is dominated by crystalline phases of GeO and TeO; this is depicted in the figure. Considering the elemental maps of as-deposited films (see supplementary materials, Table I), this measurement confirms the presence of oxides both in amorphous and crystalline states of (GeTe)_91_O_9_ and (GeTe)_88_O_12_ materials.

The (006) peak shifts to higher angle with oxygen incorporation, resulting in the compression of lattice parameter *c*. Eventually, this peak evolves toward a GeO_2_ preferred phase as the oxygen content is increased up to 6 at.%. Furthermore, the diffraction intensity of (220) peak weakens with the increasing proportion oxygen. The full-width at half-maximum of the peak is therefore increased with oxygen content, which, according to Scherrer’s equation, implies a reduction in the GeTe-O grain size. This indicates the incorporated oxygen atoms probably exist at interstitial sites and grain boundaries in the crystalline phase, suppressing the crystal growth in the material. The accumulation of dopants at grain boundaries has been widely reported in the crystalline Te-based phase change materials with light element (carbon, nitrogen, and oxygen) doping^6^,^13^,^24^,^25^.

**Figure 3:**
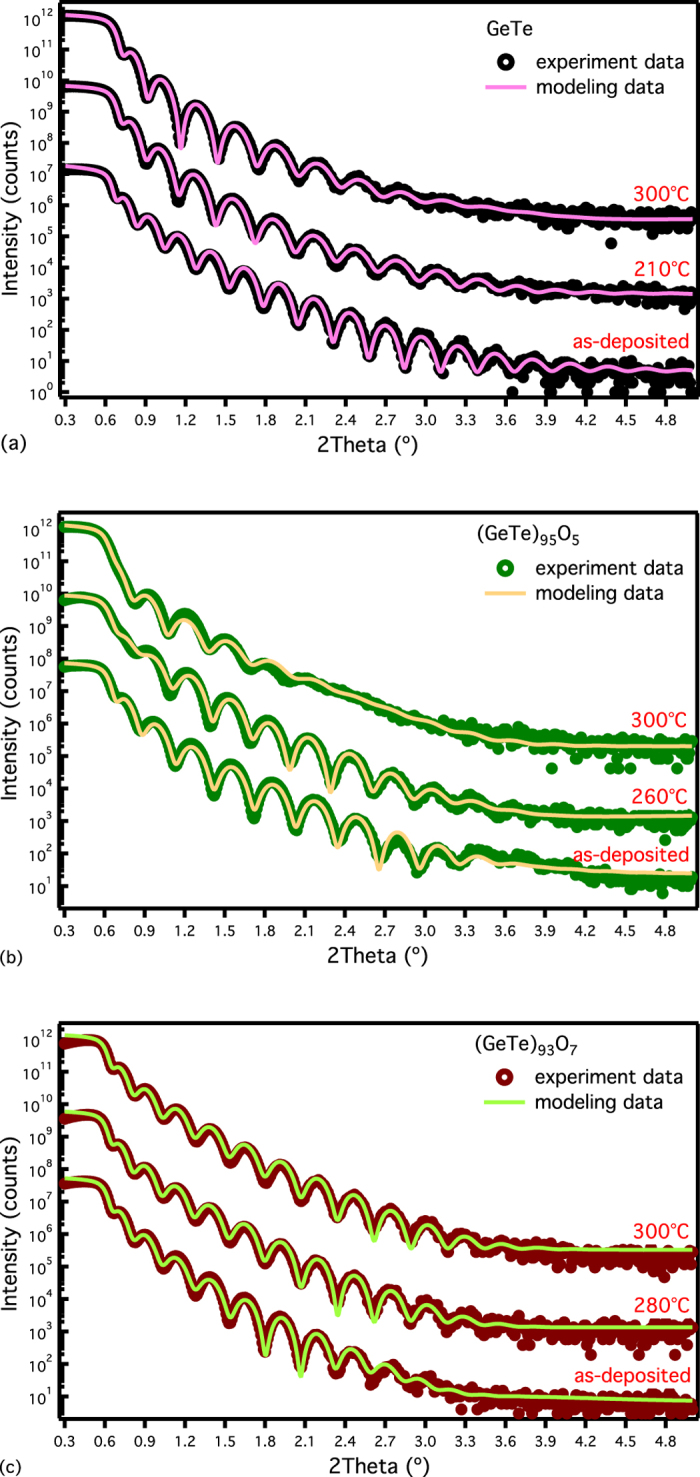
XRR scans and the corresponding modelling curves for GeTe-O films in the amorphous phase, crystalline phase after annealing at (*T*_*x*_ + 20) °C, and after annealing at 300 °C. (**a**) pure GeTe, (**b**) (GeTe)_95_O_5_, and (**c**) (GeTe)_93_O_7_ films. To aid comparison, a vertical offset is added to each crystalline curve.

Moderate oxygen incorporation increases the stability of GeTe-O. Room temperature x-ray reflectivity (XRR) measurements were performed on GeTe-O in the as-deposited amorphous state, crystallised state [crystallised at (*T*_*x*_ + 20) °C ], and after annealing at 300 °C. See Fig. 3(a–c) for GeTe, (GeTe)_95_O_5_, and (GeTe)_93_O_7_ materials, respectively. An XRR model was fit to the experimental data and used to determine the surface roughness and thickness of the films. The surface roughness of the crystalline GeTe and (GeTe)_95_O_5_ film increases significantly by crystallising as indicated from the reduction of the number of Kiessig fringes, i.e., the Kiessig fringes are smeared due to the increased surface roughness. In contrast, the surface roughness of the crystalline (GeTe)_93_O_7_ film is clearly reduced with pronounced Kiessig fringes in the 300 °C annealed curve. This also implies a smaller grain size and thus higher thermal stability as suggested above. A similar improvement in surface roughness has also been found in nitrogen doped PCMs^31^,^32^. In contrast, the loss of Kiessig fringes in the as-deposited GeTe-O films indicates increased surface roughness with O incorporation. We suspect that O is partially going into the grain boundaries and exerting bi-axial strain on the crystal and this influences the growth direction of the material. Indeed we saw in Fig. 2 that the (220) diffraction peak is suppressed, implying tensile strain in the *a* and *b* directions. The doping element induced strain field in the film can be also observed in nitrogen-doped Ge_2_Sb_2_Te_5_ material^33^.

**Figure 4:**
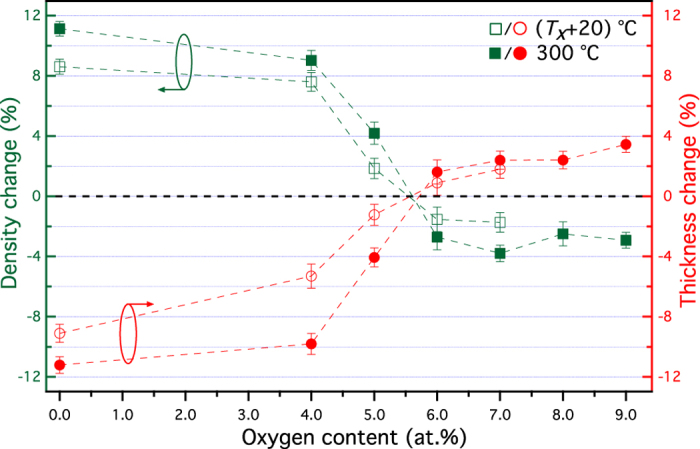
The change in film thickness (red) and mass density (green) upon crystallisation as a function of oxygen content. The films were annealed at (*T*_*x*_ + 20) °C (open symbols) and 300 °C (solid symbols).

A shift of the critical angle for total external reflection and Kiessig oscillations upon crystallisation is observed from the XRR patterns in Fig. 3. These are due to the change of the mass density and thickness of the film. In order to satisfactorily fit the modelled XRR pattern to the measured data, it was necessary to include a thin silicon oxide (~2 nm) layer between the phase change film and the Si substrate (see supplementary material, Table II). Figure 4 shows the film thickness and average mass density change upon crystallisation as a function of oxygen content. The GeTe film exhibits a thickness decrease of 9.1 ± 0.6% and the associated mass density increase of 8.6 ± 0.5% upon crystallisation (crystallised at 210 °C), which is similar to the literature value (GeTe, ~8.7% density increase^34^). Introducing oxygen into amorphous GeTe films reduces the change of film thickness and mass density upon crystallisation, as plotted in Fig. 4. The decrease in films thickness and correlated increase in mass density after 260 °C annealing is reduced to within 2% for (GeTe)_95_O_5_. At 5.5 at.% oxygen, the amorphous and crystalline states have the same density. Further increasing the oxygen to over 6 at.%, results in an unusual behaviour upon crystallisation where the mass density decreases. These effects are clear in Fig. 4, which shows the change in film thickness and mass density as a function of oxygen content.

## Discussion

It is noteworthy that the amorphous-crystalline mass density difference increases when the films are further annealed at higher temperatures. This indicates a continuous structure deformation with temperature. The decrease in mass density upon crystallisation anomaly, which was observed for films with more than 6 at.% oxygen is by no means common, and there are only a few reports of similar effects in the literature: Ge-rich Ge-Sb^35^, Sb-rich Ga-Sb^36^, and Cu-Ge-Te^37^ alloys.

The peculiar behaviour upon crystallisation of GeTe-O material can be attributed to the preferential formation of oxides (GeO_2_ and TeO_2_).The overall volume change of GeTe-O films is controlled by the shrinkage resulting from GeTe crystallisation and the thermal expansion of oxides. Simply increasing the oxygen content in the film creates smaller thickness reduction upon crystallisation. This is due to the improved structural stability against phase transition and larger thermal dilation of amorphous oxide network in the material. The zero density change upon crystallisation is then achieved by the trade-off between these two effects. At higher oxygen doping concentrations, the thermal expansion of GeTe-O materials dominates during annealing due to the increased proportion of oxide structures, which leads to a volumetric expansion upon crystallisation as shown in Fig. 4.

The observed zero density change upon crystallisation of GeTe-O material is an attractive characteristic for PCM applications. Significant hydrostatic stress on PCM cells is caused by the large mass density change during a phase transition^38^,^39^. This leads to void formation and delamination from the PCM’s surroundings; an effect that ultimately limits the cyclability of PCM devices and results in programming failures^11^. Clearly, a PCM that exhibits a small, or even zero, density change during switching could provide a means to overcome this failure mechanism and even widen the choice of materials that can be used to enclose PCMs.

Stress within PCMs also causes a shift in *T*_*x*_ and *E*_*a*_. This can be seen in Fig. 1(c), where the surface crystallises at a different temperature to the bulk. This can result in degraded performance of PCM cells. Smaller density change and thus lower stress is desired for a robust phase transition point. Moreover, resistance drift is thought to be related to structural relaxations of the PCMs^40^. Minimising the density change during switching would also minimise the post-switch residual stress and therefore reduce the resistance drift problem. Hence, these zero density change materials provide a materials engineering approach to increase the cyclability of PCM devices whilst potentially reducing resistance drift.

We also hypothesise that despite an elevated crystallisation temperature, doping oxygen into GeTe may reduce the switching energy of GeTe-O PCM devices since the switching energy is dependent on the enthalpy change, which is reduced by the smaller volume change. These topics will be discussed in future works.

In summary, doping GeTe with up to 8 at.% oxygen improves its thermal stability without substantially changing its crystal structure. We suspect that this will provide a beneficial enhancement to the data retention time when the material is employed in a PCM memory device. However, excessive oxygen doping causes phase separation into GeO_2_ and TeO_2_, which destroys the phase change ability of the material. The change of film thickness and mass density upon crystallisation is determined to decrease with oxygen incorporation, and an unusual change in the thickness and density of GeTe-O films can be observed as the oxygen content is increased. The GeTe-O material with a composition of ~5.5 at.% oxygen can provide a zero density change between the amorphous and crystalline phases, which we suspect will be useful in the design of high performance PCM devices that exhibit superior cyclability, archival stability and a reduced resistance drift.

## Methods

GeTe and GeTe-O thin films of 60-nm-thickness were prepared on fused silica and Si (100) substrates at room temperature by RF reactive sputtering of a stoichiometric GeTe (99.99%) target under an Ar/O_2_ gas mixture. The chamber base pressure was better than 2.7 × 10^−5^ Pa and the sputtering pressure was fixed at 0.5 Pa with different O_2_ reactant flow rates and a constant Ar flow rate of 20 sccm (see supplementary materials, Table I). The different O_2_ gas flow rates of 0.13, 0.2, 0.3, 0.4, 0.45, 0.5, 0.6, and 0.8 sccm were used for sputtering, resulting in (GeTe)_96_O_4_, (GeTe)_95_O_5_, (GeTe)_94_O_6_, (GeTe)_93_O_7_, (GeTe)_93_O_7_, (GeTe)_92_O_8_, (GeTe)_91_O_9_, and (GeTe)_88_O_12_, respectively. In order to void the hysteresis behaviour that is typical during the oxygen involved reactive sputtering^41^, the flow rate of O_2_ in this work was as small as 0.13 sccm. The composition of the films was confirmed by energy dispersive x-ray spectroscopy (EDX) using samples deposited on aluminum foil, where the oxygen background composition was calibrated by the results obtained from blank aluminum foil.

The crystallisation process of GeTe-O materials was studied by *in situ* monitoring both the optical transmission and reflection of 658 nm laser light from room temperature up to 330 °C. The samples were heated in an argon atmosphere at ambient pressure with heating rates ranging from 2 to 10 °C/min. The activation energies of the amorphous-crystalline phase transition were determined from the heating rate dependent optical measurements using Kissinger’s method, where *E*_*a*_ is calculated from the plots of ln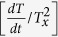 against 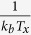
42.

Grazing incidence x-ray diffraction measurements of the crystalline films were performed at 1° grazing incidence geometry with Cu K-*α* radiation. Diffraction data were collected in 2*θ* scan mode from 20° to 55° with a step of 0.02°. The films for XRD analysis were heated at 300 °C for 20 min in an argon atmosphere.

The grazing incidence reflectivity curves in the x-ray reflectivity (XRR) experiments were measured for the GeTe-O films before and after annealing. The as-deposited amorphous films were annealed at (*T*_*x*_ + 20) °C and 300 °C under an argon atmosphere. The XRR data were recorded with 2*θ* − ω geometry in the range of 0–5°, using a step of 0.01°. A knife-edge collimator was placed very close to the sample surface to obtain sufficient intensity by blocking the direct beam. The thickness and mass density of the films were estimated by the best fitting of the experimental XRR profiles with DIFFRAC plus LEPTOS program using the Levenberg-Marquart technique. The Levenberg-Marquart algorithm is based on the code using trust-region approach^43^. The parameters of the fit are provided in the supplementary material, Table II.

## Additional Information

**How to cite this article**: Zhou, X. *et al.* A zero density change phase change memory material: GeTe-O structural characteristics upon crystallisation. *Sci. Rep.* **5**, 11150; doi: 10.1038/srep11150 (2015).

## Supplementary Material

Supplementary Information
