# Supplementary material for: A zero density change phase change memory material: GeTe-O structural characteristics upon crystallisation
Source: Sci Rep. 2015 Jun 11;5:11150. doi: 10.1038/srep11150 (PMC4464072; doi:10.1038/srep11150)
Supplement: Supplementary Information [file srep11150-s1.doc]

**A zero density change phase change memory material: GeTe-O structural characteristics upon crystallisation**

Xilin Zhou,1 Weiling Dong,1 Hao Zhang,1 and Robert E. Simpson1

1*Singapore University of Technology and Design, 8 Somapah Road, Singapore, 487372*

**SUPPLEMENTARY INFORMATION**

Measured Values of Film Composition

As summarised in Table I, the O2 gas flow rates of 0.13, 0.2, 0.3, 0.4, 0.45, 0.5, 0.6, and 0.8 sccm were employed in the reactive sputtering whilst the Ar flow rate was fixed at 20 sccm. Hence, the minimum achievable O2/Ar ratio is 0.0065, which is found effective in terms of improving the thermal stability of GeTe alloy.

The germanium, tellurium, and oxygen elemental maps shown in the last column of Table I were obtained from an EDX scan of the as-deposited GeTe-O film. At the relatively low level of oxygen doping, the overlapping elemental maps indicate a uniform distribution of the elements without any obvious composition separation. As the oxygen flow rate rises to 0.6 sccm and higher, however, some randomly distributed brighter regions are spotted in the same locations both in germanium and oxygen maps, which strongly implies the phase separation in the as-deposited material into germanium oxides.

The Parameters of XRR Fit

The best fitting of the experimental XRR profiles is performed with DIFFRAC plus LEPTOS program using the Levenberg-Marquart technique. The parameters of the XRR fit are listed in Table II.

TABLE I. The preparation conditions for GeTe-O materials in this work. Tx and Ea were measured both in transmission and reflection modes. The element maps in the last column were obtained from an EDX scan on 25 μm × 25 μm region.

| Material | O2 flow  (sccm) | O content  (at.%) | Grow rate  (Å/sec) | *Tx* (°C) | | *Ea* (eV) | | Element map | | |
| --- | --- | --- | --- | --- | --- | --- | --- | --- | --- | --- |
|  |  |  |  | *Txt* | *Txr* | *Eat* | *Ear* | Ge | Te | O |
| **GeTe** | 0 | 0 | 0.395 | 194.2 | 194.9 | 2.26 | 2.31 | 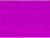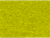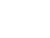 | | |
| **GeTeO0.13** | 0.13 | 4 | 0.423 | 225.2 | 226.9 | 2.32 | 2.39 | 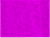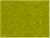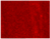 | | |
| **GeTeO0.2** | 0.2 | 5 | 0.448 | 239.7 | 242.7 | 2.42 | 2.48 | 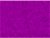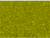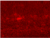 | | |
| **GeTeO0.3** | 0.3 | 6 | 0.481 | 252.1 | 255.7 | 2.72 | 2.83 | 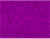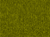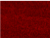 | | |
| **GeTeO0.4** | 0.4 | 7 | 0.491 | 261.1 | 265.4 | 2.96 | 2.98 | 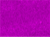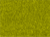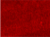 | | |
| **GeTeO0.45** | 0.45 | 7 | 0.501 | 273.2 | 278.9 | 2.86 | 2.90 | 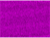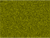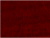 | | |
| **GeTeO0.5** | 0.5 | 8 | 0.517 | 277.8 | 279.4 | 3.59 | 3.72 | 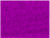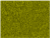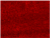 | | |
| **GeTeO0.6** | 0.6 | 9 | 0.551 | − | | − | | 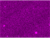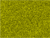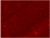 | | |
| **GeTeO0.8** | 0.8 | 12 | 0.590 | − | | − | | 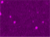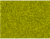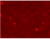 | | |

| Material | | SiO2 layer | | GeTe-O layer | |
| --- | --- | --- | --- | --- | --- |
|  |  | Thickness (nm) | Density (g/cm3) | Thickness (nm) | Density (g/cm3) |
| **GeTe** | amorphous | 1.95 | 2.65 | 31.49 | 5.46 |
|  | (*Tx*+20) °C | 2.48 | 2.41 | 28.63 | 5.93 |
|  | 300 °C | 2.34 | 2.46 | 27.96 | 6.07 |
| **(GeTe)96O4** | amorphous | 2.30 | 2.65 | 36.26 | 5.30 |
|  | (*Tx*+20) °C | 1.18 | 2.51 | 34.33 | 5.70 |
|  | 300 °C | 1.64 | 2.54 | 32.70 | 5.78 |
| **(GeTe)95O5** | amorphous | 2.15 | 2.54 | 27.99 | 5.13 |
|  | (*Tx*+20) °C | 1.89 | 2.65 | 27.65 | 5.22 |
|  | 300 °C | 2.46 | 2.34 | 26.85 | 5.34 |
| **(GeTe)94O6** | amorphous | 2.17 | 2.36 | 36.32 | 5.18 |
|  | (*Tx*+20) °C | 2.07 | 2.37 | 36.64 | 5.10 |
|  | 300 °C | 1.03 | 2.54 | 36.90 | 5.04 |
| **(GeTe)93O7** | amorphous | 1.56 | 2.65 | 31.47 | 4.98 |
|  | (*Tx*+20) °C | 1.17 | 2.39 | 32.03 | 4.89 |
|  | 300 °C | 1.89 | 2.48 | 32.22 | 4.79 |
| **(GeTe)92O8** | amorphous | 1.89 | 2.29 | 32.53 | 4.83 |
|  | 300 °C | 1.85 | 2.24 | 33.31 | 4.71 |
| **(GeTe)91O9** | amorphous | 1.70 | 2.29 | 32.52 | 4.80 |
|  | 300 °C | 1.79 | 2.43 | 33.64 | 4.66 |

TABLE II. The thickness and density values of SiO­2 and GeTe-O layers at different temperatures obtained from XRR fit.
